# Advances in sarcoma diagnostics and treatment

**DOI:** 10.18632/oncotarget.12548

**Published:** 2016-10-06

**Authors:** Amanda R Dancsok, Karama Asleh-Aburaya, Torsten O Nielsen

**Affiliations:** ^1^ Pathology and Laboratory Medicine, University of British Columbia, Vancouver, BC, Canada; ^2^ Sarcoma Disease Site Committee, Canadian Cancer Trials Group

**Keywords:** soft tissue sarcoma, sarcoma review, sarcoma diagnostics, sarcoma therapeutics, sarcoma advances

## Abstract

The heterogeneity of sarcomas with regard to molecular genesis, histology, clinical characteristics, and response to treatment makes management of these rare yet diverse neoplasms particularly challenging. This review encompasses recent developments in sarcoma diagnostics and treatment, including cytotoxic, targeted, epigenetic, and immune therapy agents. In the past year, groups internationally explored the impact of adding mandatory molecular testing to histological diagnosis, reporting some changes in diagnosis and/or management; however, the impact on outcomes could not be adequately assessed. Transcriptome sequencing techniques have brought forward new diagnostic tools for identifying fusions and/or characterizing unclassified entities. Next-generation sequencing and advanced molecular techniques were also applied to identify potential targets for directed and epigenetic therapy, where preclinical studies reported results for agents active within the receptor tyrosine kinase, mTOR, Notch, Wnt, Hedgehog, Hsp90, and MDM2 signaling networks. At the level of clinical practice, modest developments were seen for some sarcoma subtypes in conventional chemotherapy and in therapies targeting the pathways activated by various receptor tyrosine kinases. In the burgeoning field of immune therapy, sarcoma work is in its infancy; however, elaborate protocols for immune stimulation are being explored, and checkpoint blockade agents advance from preclinical models to clinical studies.

## BACKGROUND

Sarcomas are a broad family of cancers that arise from cells of mesenchymal origin in virtually every tissue of the body, and they can differentiate along a number of tissue lineages, such as adipose, muscle, fibrous, cartilage, or bone. As such, the pathology of these neoplasms is extremely diverse, with over seventy described subtypes [1]. Historically categorized as either bone or soft tissue, sarcomas are now molecularly classified into two groups: genetically complex, with a high mutational burden and a complex karyotype, or genetically simple, bearing a single disease-specific translocation, mutation, or amplification within a comparatively quiescent genomic background [2].

This histological and molecular heterogeneity makes sarcomas particularly difficult to diagnose, leading to debate surrounding the sufficiency of histological diagnosis versus the need for ancillary molecular diagnostics. Treatment has proven equally challenging, and research findings in one subtype often do not translate to others. These limitations are magnified within the context that sarcomas are among the rarest of cancer diagnoses, making research and trials more difficult. In the US, sarcomas represent 1% of new cancer diagnoses and of cancer-related deaths [3], though they are more prevalent in childhood and adolescence, where they account for 19-21% of cancer-related deaths [4]. Therefore, though the complexity of sarcomas is comparable to that of any of the more common and heavily researched malignancies, there are comparatively few novel therapeutic approaches in advanced development.

Sarcomas, as a group, are resistant to conventional cytotoxic chemotherapy, save for some successes with anthracycline-based therapy for rhabdomyosarcoma, Ewing sarcoma, and osteosarcoma [5]. Late recurrence and metastasis still occur in some subtypes, so when surgery and radiation fail, there are few - if any - effective systemic options available. Clinical trials that include sarcomas are rare and frequently confounded by lumping together results from biologically disparate subtypes, as continues to occur with molecularly divergent subcategories of liposarcoma. Given these accrual and design challenges, it can be difficult to gather convincing high-level evidence to guide the management of sarcomas.

Nonetheless, the past year has seen advances in genomics-based sarcoma science and the publication in major journals of significant positive results from clinical trials. In this review, we aim to summarize recent developments in both diagnostics and treatment, including translational science and clinical trials in chemotherapy, targeted therapy, epigenetic therapy, and the burgeoning field of immune therapy. The scope of this review includes works published from late 2014 to early 2016.

## SARCOMA DIAGNOSTICS

### Genomic landscapes in sarcoma

Multi-platform “omics” approaches were undertaken to elucidate comprehensive mutational landscapes for liposarcomas, epithelioid sarcoma, and rhabdomyosarcomas.

Kanojia et al [6] used a combination of single nucleotide polymorphism (SNP) arrays and whole- and targeted-exome sequencing to characterize the genomic landscape of 86 liposarcomas of all major subtypes. In addition to the expected amplifications in MDM2 and other known 12q amplicon genes CDK4 and HMGA2, they identified a number of novel gene amplifications: UAP1, MIR557, LAMA4, CPM, IGF2, ERBB3, and IGF1R. Of particular interest, CPM (carboxypeptidase M) - located at the edge of the 12q amplicon, outside of what was thought to be the key region defined by CDK4 and MDM2 - was amplified in 39 of 50 well- and de-differentiated liposarcomas. Knockdown of CPM reduced cell line and xenograft growth, migration, and invasion, and reduced expression of phosphorylated EGFR, Akt, and ERK, suggesting that CPM is involved in epidermal growth factor signalling, a targetable pathway that might play an unanticipated role in liposarcomagenesis. This genomic survey also found recurrent mutations in genes associated with cell adhesion, cytoskeletal organization, base excision repair, homologous recombination repair, nucleotide excision repair, and DNA replication: PLEC, MXRA5, FAT3, NF1, MDC1, TP53, and CHEK2. The NF1 (neurofibromin-1) gene was of particular interest, altered in 13 of 50 well- and de-differentiated liposarcomas. Knockdown of NF1 increased cell line proliferation and xenograft growth, suggesting a potential tumor suppressor role for this gene in keeping with its function as a regulator of Ras signalling.

Jamshidi et al [7] published the first next-generation sequencing study of epithelioid sarcoma, a rare but clinically-devastating sarcoma that typically presents in the distal extremities of young adults and does not respond to available systemic therapy. Whole genome and transcriptome sequencing on seven tumor specimens and three cell lines confirmed SMARCB1 loss by variable mechanisms, but revealed a complex genome with an unexpectedly high mutational rate for a tumor of younger patients. This high mutational burden is in direct contrast with the genomic profile of rhabdoid tumor, a pediatric neoplasm also driven by the loss of SMARCB1; the mutation rate of epithelioid sarcoma is three orders of magnitude greater than that of rhabdoid tumor.

Shern et al [8] characterized 147 tumor-normal pairs in rhabdomyosarcomas using a combination of whole- genome, exome, and transcriptome sequencing. The overall burden of somatic mutations was relatively low, but several genes were recurrently altered, including previously reported mutations in NRAS, KRAS, HRAS, FGFR4, PIK3CA, and CTNNB1, and novel mutations in FBXW7 and BCOR. Importantly, authors noted that the receptor tyrosine kinase/RAS/PIK3CA-associated networks were altered in 93% of cases, giving therapeutic implications for this disease. Tumors segregated predictably into subtypes with and without a PAX3/PAX7 gene fusion with FOXO1 or alternative partners, including a novel fusion of PAX3 with the C-terminus of INO80D. They asserted that the presence or absence of a PAX fusion more accurately describes the genomic landscape and biology of rhabdomyosarcoma than the traditional alveolar versus embryonal histology-based subtyping, and that it is a better predictor of clinical behaviour [9] and prognosis [10,11]. This brings up the familiar question as to whether the traditional morphologic diagnosis of sarcomas is adequate, or whether molecular techniques ought to be mandatory for sarcoma diagnoses.

### Molecular diagnostics

To interrogate the need for and value of ancillary molecular diagnostics for sarcomas, Italiano et al [12] designed GENSARC, a prospective study across 32 centres in France, that compared the diagnostic accuracy of histological assessment by a sarcoma subspecialty pathologist with and without molecular genetic testing. Sarcomas were accrued whenever one of six subtypes presented, based on histological diagnosis (categorized as certain, probable, or possible diagnosis): dermatofibrosarcoma protuberans (COL1A1-PDGFB translocation, leading to PDGβ overexpression), dedifferentiated liposarcoma (MDM2 amplifications), Ewing sarcoma (EWSR1 translocations), synovial sarcoma (SS18 translocations), alveolar rhabdomyosarcoma (PAX3/7 translocations), and myxoid liposarcoma (FUS-DDIT3 translocations). Expert pathologists committed to a diagnosis, level of certainty, and differential diagnosis prior to completion of molecular tests. Comparative molecular testing included fluorescence in-situ hybridisation (FISH) and/or quantitative or reverse transcriptase polymerase chain reaction. Of 384 cases, molecular testing resulted in a change in diagnosis in 53 (14%) cases, leading to a change in management or prognosis in 45 (12%) cases. Based on these findings, the authors recommend mandatory molecular testing - even when the histological diagnosis was made by a sarcoma subspecialty pathologist - for accurate diagnosis and appropriate clinical management of sarcoma. However, it is important to note that almost no cases where the pathologist was “certain” had their diagnosis change as a result of molecular testing. Cases that were “probable” or “possible” had frequent diagnostic changes, but these are cases where pathologist will almost always order molecular ancillary testing if available, or refer out for testing if not.

Regardless of the necessity for molecular testing to support certain diagnoses, detailed genetic analysis of a patient's tumor tissue can have important implications for targeted therapy. Studies at many institutions are applying next-generation sequencing strategies in an attempt to identify “actionable” mutations with some clinical significance to cancer, sarcoma centres included. Peds-MiOncoSeq [13] employed exome and transcriptome sequencing in 91 pediatric and young adult cancers, including 25 sarcomas. “Potentially actionable findings” were identified in 42 (46%) cases, leading to an actual change in treatment for 14 (15%) patients. This included one rhabdomyosarcoma patient, whose diagnosis and treatment plan changed following sequencing results, and who remained in remission 6 months after the change in management. This study lacked any control group, so it is not possible to assess whether outcomes improved compared to standard care. A similar study, iCat [14], was an “individualized Cancer therapy” effort in advanced pediatric solid tumors. Profiling included targeted DNA sequencing and copy number assessment. Of the 89 patient tumors profiled - including 12 Ewing sarcomas, 11 osteosarcomas, 11 rhabdomyosarcomas, and 27 other soft tissue sarcomas - 43% had clinical implications: an actionable mutation leading to an FDA-cleared or open clinical trial targeted therapy, a translocation that changed diagnosis, or the identification of an underlying cancer predisposition syndrome. Three patients received matched therapy. Chang et al [15] combined whole exome and transcriptome sequencing with SNP arrays in 59 relapsed and refractory pediatric and young adult patients, including 29 sarcomas. Thirty (51%) had clinically actionable alterations. Actionability included somatic mutations targetable by available therapies (41%), change in diagnosis (12%), or reportable germline mutation for which patients received family genetic counselling (12%).

Outside of the clinic, several more studies searched for actionable/targetable and recurrent/driver mutations across sarcomas. Andersson et al [16] conducted a next-generation sequencing panel of 207 hotspots in 50 cancer-associated genes, in Ewing sarcomas (n = 22), synovial sarcomas (n = 14), gastrointestinal stromal tumors (GIST; n = 9), myxoid liposarcomas (n = 7), and Ewing-like small round cell tumors (n = 3). They identified mutations in 8 driver genes in Ewing sarcoma (NRAS, MET, HRAS), Ewing-like small round cell tumors (BRAF, SMARCB1), GIST (KIT, PDGFRA), and synovial sarcoma (CTNNB1). The BASIC3 study by Parsons et al [17] explored the diagnostic yield of whole-exome sequencing in 121 pediatric solid tumors, including 9 rhabdomyosarcomas, 6 Ewing sarcomas, 4 osteosarcomas, and 7 other soft tissue sarcomas. Nearly 40% yielded actionable mutations, in the form of somatic mutations of established clinical utility (3%) or potential clinical utility (24%), or in diagnostic germline findings related to patient phenotype (10%). CTNNB1 was the most frequently mutated somatic gene, plus KIT, TSC2, and MAPK pathway genes (BRAF, KRAS, NRAS). Movva et al [18] had access to 2539 sarcoma specimens, encompassing 61 bone and soft tissue sarcoma subtypes. Up to 2434 samples were profiled by immunohistochemistry, 1048 by FISH, 591 by next-generation sequencing, and 1250 by Sanger sequencing. By immunohistochemistry, they noted overexpression of TOPO2A in 52.8% of cases, SPARC in 35.9%, and PDGFRA in 22.1%. Low expression of MGMT was noted in 65.3% of cases, and loss of PTEN was seen in 38.6%. By DNA sequencing, the most commonly mutated genes were TP53 (26.3%) and BRCA2 (17.6%). Dual TOPO2A overexpression by immunohistochemistry and TP53 DNA mutation was observed in 85.8% of samples. As study cohorts were not consistently defined, and methodologies were not consistently applied, the results from this study are best considered hypothesis-generating, in need of further validation.

Returning to the question of the necessity of comprehensive molecular analysis for the diagnosis and management of sarcomas, we conclude that the evidence at this point is not strong enough to support mandatory use of these expensive and work-intensive diagnostic tools. As of yet, there has been no significant clinical impact reported in terms of treatment response or patient survival. While comprehensive genomics may certainly prove useful in complex cases, it may be most practical for the use of these tools to remain at the discretion of the sarcoma subspecialty pathologist. In the setting of routine clinical practice, traditional morphological assessment (based on H&E slides, supplemented by immunohistochemistry) is likely sufficient for most sarcoma diagnoses.

### Fusion identification

Next-generation sequencing of RNA opens the door to identify both known and novel fusion transcripts. Hofvander et al [19] applied massively parallel paired-end mRNA sequencing to 8 sarcomas, including 2 osteosarcomas, 2 myxofibrosarcomas, 1 low-grade fibromyxoid sarcoma, 1 fibrosarcoma, 1 undifferentiated pleomorphic sarcoma, 1 glomus tumor, and 1 myxoid liposarcoma. By this method, they identified two known fusions (FUS-CREB3L2 and HAS2-PLAG1) and three novel fusions (KIAA2026-NUDT11, CCBL1-ARL1, and AFF3-PHF1), highlighting this technique as an effective strategy for fusion detection. The myxoid liposarcoma was reclassified as a lipoblastoma due to absence of the pathognomonic myxoid liposarcoma FUS-DDIT3 fusion and presence of a HAS2-PLAG1 fusion. Authors note that sequencing data was analysed by three different algorithms, but only two of the fusions were reported by more than one software program. They therefore recommend use of more than one algorithm to analyze sequencing output.

This type of approach may be useful to assist in identifying molecular events that can be useful for the differential diagnosis of some challenging sarcoma categories, such as vascular tumors of bone. Epithelioid hemangioma of bone can be difficult to differentiate from other vascular neoplasms due to its highly variable histological presentation. Ijzendoorn et al [20] identified a balanced t(3;14) translocation in an index case, and transcriptome sequencing revealed that the translocation involved a break in exon 4 of the FOS proto-oncogene, leading to introduction of a stop codon and loss of its transactivation domain. Fusions generating FOS truncations were observed in 5 of 7 samples of epithelioid hemangioma (with variable fusion partners). This finding proffers a new mechanism of tumorigenesis and a potentially useful diagnostic tool and treatment target for epithelioid hemangioma of bone.

### New sarcoma classification

RNA sequencing has also proven useful in defining new entities among otherwise unclassifiable sarcomas. Le Loarer et al [21] conducted RNA sequencing on 32 round-cell sarcomas which did not fit into known specific diagnostic categories, and they identified 4 index cases bearing mutations in SMARCA4. Postulating that the partially rhabdoid morphology seen in these cases reflects underlying BAF complex inactivation, they performed a targeted sequencing screen on 18 unclassified sarcomas with partial rhabdoid phenotypes, noting SMARCA4 mutations exclusively in the 6 thoracic tumors of the cohort. After finding 9 additional cases (based on inferred characteristics of the initial cohort), they identified a total of 19 samples with SMARCA4 mutations. By whole transcriptome sequencing, these samples clustered apart from the other unclassified sarcoma samples, defining a new entity of aggressive, poor prognosis thoracic primary sarcomas of young adults: “SMARCA4-deficient thoracic sarcomas.” Comparison to the profiles other SMARCA4-deficient malignancies showed that these are distinct from lung carcinomas but related to malignant rhabdoid tumors and to small-cell carcinoma of the ovary, hypercalcemic type.

## CHEMOTHERAPY

### The treatment paradigm of soft tissue sarcoma with chemotherapy

While basic and translational research delve deeper into tumor biology to identify new strategies to target these multifaceted malignancies, conventional chemotherapy remains a mainstay in the treatment of a number of sarcomas. Indeed, this was the subject of many major findings over the past year.

For patients with primary soft tissue sarcomas, surgery with or without radiotherapy can offer a cure, but nearly half of patients recur and eventually die, with an estimated median survival of 12 to 15 months [22]. As a result, treatment of metastatic or unresectable disease with cytotoxic agents is often given for palliative rather than curative purposes [23]. These cytotoxic agents often incorporate anthracycline- or gemcitabine-based regimens as a first line treatment [24–27]. Other agents such as dacarbazine and ifosfamide, only show clinical improvement in overall response rate and progression free survival (PFS), without significant benefit in overall survival [22,28–31]. Moreover, despite superior PFS observed with these conventional cytotoxic therapies, they are fraught with severe toxicities and attendant high costs, a burden for both patients and health care systems. Currently, there is no consensus standard of care for chemotherapy regimens in metastatic sarcoma patients, in part due to treatment strategies that were developed somewhat empirically, rather than through specific, rational targeting of molecular subtype and/or pathogenic mechanism. Consequently, research efforts are underway to address the value of chemotherapy in sarcoma subtypes, as well as to investigate different and newer systemic therapies. Recently published trials have demonstrated promising positive results using newer cytotoxic agents, including eribulin, trabectedin, and aldoxorubicin. Table 1 summarizes the clinical trials in cytotoxic chemotherapy described below.

**Table 1:** A survey of recently published chemotherapy clinical trials in sarcoma

Regimen	Trial Analyzed	Design	Stage	Subtype	N	Median PFS (months)	Median OS (months)
Eribulin vs. Dacarbazine	Schoffski et al. Lancet. 2016	Phase III	Advanced	Liposarcoma LMS	452	2.6 in both arms (NS)	13.5 vs. 11.5
Trabectedin vs. Dacarbazine	Demetri et al. JCO. 2016	Phase III	Advanced	Liposarcoma LMS	518	4.2 vs. 1.5	12.4 vs. 12.9 (NS)
Trabectedin vs. BSC	Kawai et al. Lancet Oncol. 2015	Phase II	Advanced	Translocation-associated sarcoma	76	5.6 vs. 0.9	not reached vs. 8 months
Trabectedin with doxorubicin	Pavtier et al. Lancet Oncol. 2015	Phase II	Advanced	Uterine and soft tissue LMS	109	8.2 in uterine LMS vs. 12.9 in soft tissue LMS	20.2 uterine LMS vs. 34.5 in soft tissue LMS
Aldoxorubicin vs. Doxorubicin	Chawla et al. JAMA Oncol. 2015	Phase IIb	Advanced	STS	123	5.6 vs. 2.7	15.8 vs. 14.3
Gemcitabine-docetaxel	Seddon et al. Clin Sarcoma Res. 2015	Phase II	Advanced	LMS	44	7.1	17.9
Gemcitabine-docetaxel-bevacizumab	Dickson et al. Sarcoma. 2015	Phase II	Advanced	LMS UPS Pleomorphic liposarcoma Angiosarcoma	35	Not reached (3 months PFS=76%)	Not reached
Doxorubicin-ifosfamide vs. Gemcitabine-docetaxel	Davis et al. Eur J Cancer. 2015	Phase II	Early stage	STS	80	37 vs. Not reached (NS)	Not reached

Abbreviations: PFS = progression-free survival, OS = overall survival, LMS = leiomyosarcoma, STS = soft tissue sarcoma, UPS = undifferentiated pleomorphic sarcoma, NS = not significant

### Clinical trials

#### Eribulin

Eribulin is an analogue of the marine-derived compound halichondrin B that acts as an inhibitor of microtubule dynamics through binding to a single site on tubulin, thereby suppressing the stability and growth of microtubules [32,33]. Eribulin has been shown to promote apoptosis, to suppress migration and invasion of cancer cells, and to induce vascular remodeling [33–35].

The role of eribulin was evaluated in a phase III trial that randomized patients with advanced leiomyosarcomas or liposarcomas to receive either eribulin or dacarbazine [36]. Eligible patients had received at least two previous systemic regimens for advanced disease, including anthracyclines. In this trial, patients treated with eribulin demonstrated a significant improvement of two months for the study's primary endpoint of overall survival (OS), compared to the dacarbazine standard-of-care arm. However, no significant differences were observed in the secondary endpoint of PFS, and patients experienced a higher toxicity rate of 67% grade 3+ adverse events when treated with eribulin, versus 56% in the dacarbazine arm. Importantly, a pre-planned exploratory subgroup analysis showed that the treatment effects of eribulin were limited to patients with liposarcoma (who had a 7-month improvement in OS), whereas no evidence for eribulin efficacy was observed in the leiomyosarcoma subgroup. Based on this analysis, eribulin was approved in early 2016 by the US Food and Drug Administration (FDA) for metastatic liposarcoma patients. It should be noted that the results of the survival analysis in liposarcomas were not broken down into their three molecularly-distinct subtypes (i.e., dedifferentiated, myxoid, and pleomorphic liposarcoma) due to power concerns [36].

### Trabectedin

Trabectedin is a marine-derived drug that has several anti-cancer mechanisms of action [37]. It acts mainly through binding to the minor groove of DNA, inhibiting DNA binding proteins - including transcription and DNA repair complexes - ultimately leading to disruption in cell cycle and induction of apoptosis [37,38].

A phase III trial assessed the role of trabectedin versus dacarbazine in a similar cohort to that included in the aforementioned eribulin trial, again including metastatic leiomyosarcoma or liposarcoma patients who had received at least one prior systemic therapy in addition to anthracyclines [39]. This trial showed a significant improvement of 3.7 months in the secondary endpoint of PFS, favoring trabectedin over dacarbazine, while no statistically significant results were observed for the primary endpoint of OS. Trabectedin PFS benefit was observed across all pre-planned subgroup analyses including different histology subtypes. This data led to trabectedin's approval by the FDA in October 2015 for use in metastatic liposarcoma and leiomyosarcoma, indications that had already been approved in Europe. Interestingly, the clinical outcomes reported in this study contrast somewhat with those observed in the eribulin trial - which accrued patients with similar inclusion criteria and characteristics - again reporting higher rates of toxicity in the trabectedin arm than with the standard therapy of dacarbazine. Overall, the results reported in these clinical trials do not explicitly support a preferred regimen of either eribulin or trabectedin in the metastatic setting of liposarcoma, but the differences observed in clinical outcomes might reflect differences in the mechanisms-of-action of trabectedin versus eribulin that should be further investigated.

The role of trabectedin as a modulator of the transcription of oncogenic fusion proteins was demonstrated in a phase II trial assessing the efficacy and safety of trabectedin as a second line (or later) therapy for patients with advanced translocation-associated sarcomas [40]. Patients included in this trial had mainly myxoid liposarcoma and synovial sarcoma and were randomized to receive either trabectedin or best supportive care. This trial showed a statistically significant 5-month advantage in the primary endpoint of PFS with trabectedin administration [40]. Importantly, in a subsequent evaluation of 30 patients who crossed over to trabectedin after disease progression on best supportive care, trabectedin was still effective in improving PFS [41].

Beyond the established activity of trabectedin as second-line therapy for sarcomas, its role in the first-line setting was examined in a phase II single-arm clinical trial using a combination of trabectedin and doxorubicin [42]. Among 108 assessable patients with leiomyosarcoma of the uterus or soft tissue, nearly 90% achieved disease control [42]. While this trial showed promising results regarding the combination of trabectedin and doxorubicin in the first-line setting of leiomyosarcoma, a previous study by the European Organization for Research and Treatment of Cancer (EORTC) and Sarcoma Alliance for Research through Collaboration (SARC) groups, comparing trabectedin versus doxorubicin as a first-line therapy in advanced soft tissue sarcoma, did not show any superiority for trabectedin over doxorubicin [43]. Accordingly, the combination of trabectedin and doxorubicin needs to be assessed head-to-head against the effective standard-of-care control arm of doxorubicin monotherapy or doxorubicin-ifosphamide.

### Aldoxorubicin

Aldoxorubicin is a novel pro-drug of doxorubicin, where a pH-sensitive linker conjugates doxorubicin to albumin. The exposure of the albumin-drug conjugate to the acidic tumor microenvironment releases doxorubicin, which preferentially localizes in tumor cells that are pinocytotically-active [44,45]. Drug uptake within tumor tissues is further enhanced through defective lymphatic drainage and high permeability, promoting macromolecule retention in tumor tissues [46]. The net result is a capacity to deliver a higher dose of doxorubicin into tumor cells than is received by normal cells (e.g., myocardium), improving the therapeutic index.

Superior clinical activity of aldoxorubicin was recently demonstrated in a phase IIb study that showed a significantly greater PFS (the primary endpoint), in patients with advanced soft tissue sarcoma randomized to receive aldoxorubicin or doxorubicin [47]. However, no significant differences were observed in OS. Currently, the safety and efficacy of aldoxorubicin is being assessed in combination with ifosfamide (phase I/II trial, NCT02235701) or in combination with gemcitabine (phase I trial, NCT02235688) in metastatic soft tissue sarcoma patients. In addition, aldoxorubicin is being investigated in a phase III trial versus investigator's choice of treatment in metastatic soft tissue sarcomas (NCT02049905).

### Gemcitabine and docetaxel

Gemcitabine and docetaxel combinations have previously displayed a proven activity, particularly in advanced leiomyosarcoma [48]. These results have recently led to the design of a phase III trial comparing gemcitabine-docetaxel versus the conventional therapy of doxorubicin in the first-line setting of metastatic soft tissue sarcoma (GeDDis) [48]. This trial did not report significant differences in OS or PFS between the two treatment arms (data not yet published). Furthermore, the gemcitabine-docetaxel combination was stopped early due to toxicity, and thus, doxorubicin was recommended to remain as the standard first-line treatment for metastatic soft tissue sarcoma [48].

Beyond its role in the metastatic setting, the gemcitabine-docetaxel combination was recently assessed in early-stage soft tissue sarcomas. A phase II trial compared the combination of gemcitabine-docetaxel against conventional therapy with doxorubicin-ifosfamide in patients with localized, resectable, high-risk sarcoma. While this trial showed that the gemcitabine-docetaxel combination was tolerable, it did not show a significant superiority of gemcitabine-docetaxel over doxorubicin-ifosfamide in hospitalization rate, which was (somewhat unusually) defined as the primary endpoint of this study [49].

Further phase II trials have also investigated whether the activity of gemcitabine and docetaxel is enhanced by the addition of anti-angiogenic agents such as bevacizumab [50], particularly as targeted therapy for vascular sarcomas [51]. While these studies reported only modest response rates, more than half of the patients achieved stable disease. In an attempt to appropriately evaluate these findings in a phase III trial, a double-blind, placebo-controlled, randomized trial of gemcitabine and docetaxel with or without bevacizumab was designed [26]. Eligible patients had a diagnosis of leiomyosarcoma, undifferentiated pleomorphic sarcoma, pleomorphic liposarcoma, or angiosarcoma. However, due to a slow recruitment rate, the trial was changed to a single-arm study evaluating only the addition of bevacizumab to gemcitabine and docetaxel, reporting a PFS of 76% at 3 months [26]. While this PFS rate is impressive and higher than expected from similar regimens without bevacizumab [52], the absence of a control arm - a common problem in sarcoma trials - makes it hard to achieve a high level of evidence supporting the role of bevacizumab in advanced soft tissue sarcomas. These findings highlight the overall need for more collaborative, randomized phase III clinical trials that could confirm benefits suggested by single-arm phase II studies.

## TARGETED THERAPY

### Translational science

**Figure 1:**
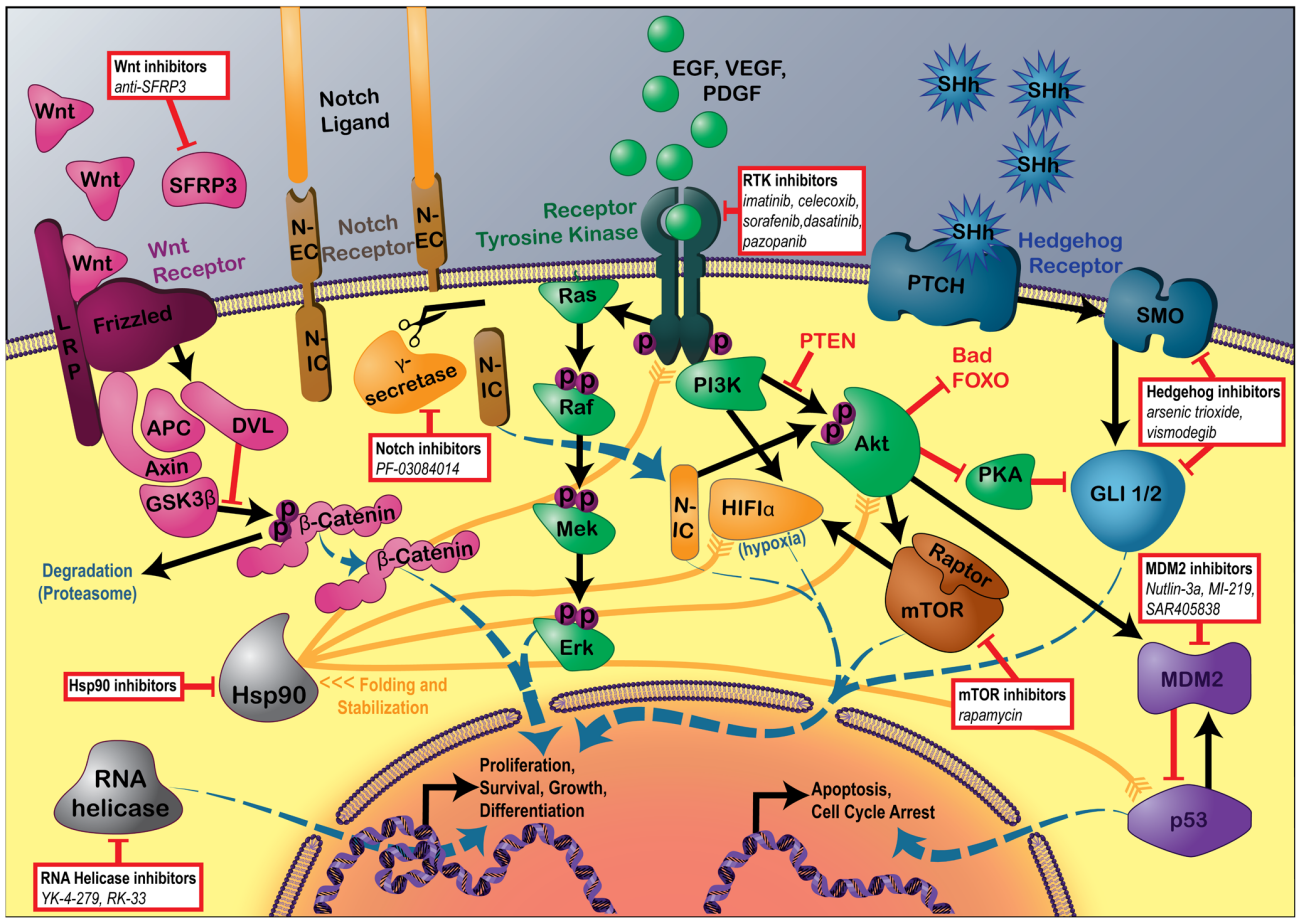
Signaling pathways currently targeted in sarcoma translational research Details of Wnt, Notch, Receptor Tyrosine Kinase, Hedgehog, MDM2, and mTOR signaling pathways, including common regulators and pathway interactions. Hsp90 protein clients also depicted. Boxes indicate the specific agents described in this review that have seen progress in the past year.

Basic science studies have identified a number of targetable oncogenic pathways activated in specific sarcomas. Figure 1 depicts the agents and targeted molecules discussed below, including regulators and cross talk between the pathways.

### Receptor tyrosine kinases

Evidence behind receptor tyrosine kinase inhibitors in sarcomas is comparatively advanced, with a number of agents in clinical trials. Aside from gastrointestinal stromal tumors, there is little known about biomarkers predictive for response. A phase II study of imatinib in 33 progressive, unresectable desmoid tumors [53] assessed the correlation between CTNNB1 (β-catenin) mutation status and “progression arrest rate” - the proportion of patients showing complete or partial response, or stable disease by Response Evaluation Criteria in Solid Tumors (RECIST) version 1.0 - 6 months after initiation of imatinib treatment. CTNNB1 mutations are found in 80% of desmoid tumors, either at threonine 41 (T41) or serine 45 (S45), the latter correlating with increased risk of recurrence [54–56]. Progression arrest rate was highest in patients bearing the S45 mutation, at 85%. The next best response was seen in patients with the T41 mutation (70%), followed by those with wild-type CTNNB1 (43%). According to this study, CTNNB1 mutation status may be predictive of response to imatinib; however, given that wild-type CTNNB1 is generally associated with better prognosis [54–56], this study design may have selected for a subpopulation of more clinically aggressive wild-type desmoid tumors. Furthermore, desmoid tumors frequently undergo spontaneous progression arrest, and clinical studies of this vexatious neoplasm really require control arms if improved progression arrest is to be attributed to kinase inhibitors or other systemic interventions [57].

In pre-clinical studies of osteosarcoma, Liu et al [58] show synergy between kinase inhibitor ZD6474 and non-steroidal anti-inflammatory agent celecoxib in cell lines and mouse xenografts. ZD6474, a small-molecule inhibitor of VEGFR-2 and EGFR, inhibited the proliferation of three osteosarcoma cell lines, promoting cell cycle arrest and apoptosis. Based on high COX-2 expression in osteosarcoma cell lines, authors also tested the COX-2 inhibitor celecoxib in vitro and saw a similar anti-proliferative effect. Combination treatment of ZD6474 and celecoxib demonstrated synergistic effects in one cell line and additive effects in the other two. Synergy between the two agents was also observed in cell line xenografts.

### mTOR

Malignant peripheral nerve sheath tumors (MPNSTs) have been shown to have receptor tyrosine kinase gene amplifications of PDGFR and cKIT [59]; however, imatinib did not show promise in a phase II trial [60]. mTOR is a key member of the PI3K/AKT axis of RTK signalling, so combining mTOR and RTK inhibition may reduce opportunities for tumor escape and therapeutic resistance. In a study comparing two kinase inhibitors plus or minus mTOR inhibitor rapamycin [61], combination treatment enhanced the anti-proliferative effects against an MPNST cell line from 40-45% per single agent to 70% inhibition of cell proliferation. In a mouse xenograft, addition of rapamycin to kinase inhibitors resulted in enhanced tumor suppression, and following cessation of treatment, reduced tumor regrowth.

### Notch

Shang et al [62] observed that nuclear Hes1, a downstream effector of Notch, is highly expressed in desmoid tumor cell lines compared to dermal scar tissue. Gamma-secretase inhibitors inhibit Notch signalling by blocking cleavage of Notch's intracellular domain, NICD. A recent phase I trial of γ-secretase inhibitor PF-03084014 in desmoid tumors [63] showed promising results, leading to the initiation of an ongoing phase II trial (NCT01981551) [64]. To further evaluate this agent's activity in desmoid tumors, Shang et al [62] exposed a panel of desmoid tumor cell lines to PF-03084014. Treatment inhibited cell growth and decreased migration and invasion. There was also a drop in NCID and expression of Hes1 in exposed cell lines. Taken together, these studies suggest that Notch signalling is important for desmoid tumor survival and that PF-03084014 is a worthwhile strategy to investigate for the treatment of desmoid tumors.

### Wnt

In a rhabdomyosarcoma study aiming to identify targetable proteins associated with alveolar rhabdomyosarcoma fusion oncoprotein [65], microarray expression profiling was performed on PAX3-FOXO1-expressing human skeletal muscle myoblasts. Transcriptome analysis revealed alteration of Wnt pathway gene members, including SFRP3 (secreted frizzled related protein 3). Knockdown of SFRP3 in an alveolar rhabdomyosarcoma cell line led to reduced cell growth and proliferation, cell cycle arrest, and apoptosis. In a conditional alveolar rhabdomyosarcoma murine xenograft system, suppression of SFRP3 reduced tumor growth and increased myogenic differentiation. Combining SFRP3 suppression with chemotherapy agent vincristine was more effective at reducing alveolar rhabdomyosarcoma cell line growth than either treatment alone, and the addition of vincristine ablated tumorigenesis in the conditional murine xenograft.

### Hedgehog

Hedgehog pathway inhibitors show promise in osteosarcoma, through targeting of SMO [66] or GLI family [67,68] hedgehog transcription factors. Saitoh et al [69] examined hedgehog inhibitors arsenic trioxide and vismodegib in combination with conventional chemotherapy agents cisplatin, ifosfamide, or doxorubicin in cell-line and xenografts. In vitro and in vivo, osteosarcoma cell proliferation and tumor growth were inhibited by any pairing of a Hedgehog pathway inhibitor with a standard chemotherapy agent. Combinations were designated as synergistic by combination-index analyses.

### Hsp90

Hsp90 is a chaperone protein that assists in the folding of many of the signalling molecules mentioned above. In a study of undifferentiated pleomorphic sarcoma [70], Hsp90 was found to be highly expressed in 56.4% of cases and was associated with poor prognosis. Expression correlated with p-Akt, p-mTOR and p-S6RP, potentially implicating Hsp90 in the activation of the mTOR pathway. In vitro, inhibition of Hsp90 decreased cell viability and inactivated the mTOR pathway. In a study by Ernst et al [71] expression of HSP90 was found to be associated with radioresistance by comparing transcriptomes of sarcoma cell lines with radioresistance scores (generated by principal component analysis). Expression of HSP90 strongly correlated with high radioresistance scores, and subsequent HSP90 inhibition sensitized radioresistant sarcoma cell lines to radiation therapy. Hsp90 inhibition was also shown to help overcome kinase inhibitor resistance in myxoid liposarcoma [72], contributing to rapid cell death in cell lines and necrosis in cell line xenografts.

### MDM2

MDM2 is a suppressor of genome guardian p53, and its genetic amplification is a hallmark of well- and de-differentiated liposarcoma. Bill et al [73] compared the effectiveness of MDM2 inhibitors Nutlin-3a, MI-219, and novel agent SAR405838. In vitro, all three agents effectively reactivated the p53 pathway, impeding cell proliferation and inducing cell-cycle arrest and apoptosis. The highest potency was seen with SAR405838, and this antitumor effect persisted in treatment of dedifferentiated liposarcoma mouse xenografts. Another study on MDM2 inhibitor Nutlin-3 in DDLPS [74] found that this agent was only effective in cell lines with wild-type p53. Nutlin-3 had little effect on p53-deficient cell lines, presenting p53 mutation as a mechanism of MDM2 inhibitor resistance. In these cell lines, addition of histone deacetylase (HDAC) inhibitor treatment overcame Nutlin-3 resistance, and was associated with PTEN and p21 up-regulation and inactivation of AKT.

### RNA helicase

FLI1, the most common translocation partner of EWSR1 in the oncogenic fusion protein driving Ewing sarcoma, requires binding to transcriptional cofactor RNA Helicase A for full activity. YK-4-279, a small molecule that blocks the interaction between FLI1 and RNA helicase A, was tested in a Ewing sarcoma cell line xenograft [75] and induced tumor regression with daily dosing. A related RNA helicase, DDX3, was found to be highly expressed in a number of sarcoma subtypes [76]. Suppression of DDX3 by knockdown or by its small-molecule inhibitor RK-33 was cytotoxic to Ewing sarcoma cell lines, and in a patient-derived Ewing sarcoma xenograft, RK-33 inhibited tumor growth without evident toxicity. Taken together, these studies support the development of RNA helicase inhibition as a new targeted strategy for Ewing sarcoma.

### FOXM1

FOXM1 is a transcription factor - sometimes activated as part of the Wnt or Hedgehog pathways - that contributes to proliferation and cell cycle progression. It has been shown to be overexpressed in numerous sarcomas [77,78], which Eisinger-Mathason et al. [77] claim is attributable to deregulation of the Hippo pathway, based on copy number variation analysis of The Cancer Genome Atlas sarcoma database (mostly leiomyosarcoma, dedifferentiated liposarcoma, myxofibrosarcoma, and UPS). In fact, they postulate that overexpression of FOXM1 by Hippo effector protein YAP is necessary for tumorigenesis in some sarcomas, based on reduced proliferation seen in a mouse model of UPS after YAP knockdown. Knockdown or pharmacological (thiostrepton) inhibition of FOXM1 itself also impaired proliferation and reduced tumor size in the UPS mouse model, a finding which was also shown in synovial sarcoma cell lines by Maekawa et al [78]. Together, these findings highlight FOXM1 as a potential therapeutic target for some sarcomas.

### Clinical trials

While significant advancements have been reported in clinical trials of cytotoxic chemotherapeutic agents, metastatic soft tissue sarcomas still have poor outcomes and few effective therapeutic options. Table 2 summarizes the clinical trials in targeted therapy described below.

**Table 2:** A survey of recently published targeted therapy studies in sarcoma

Targeted Therapy	Study Analyzed	Design	Stage	Subtype	N	Median PFS (months)	Median OS (months)
Imatinib	Stacchiotti et al. Clin Cancer Res. 2016	retrospective	Advanced	DFSP	10	11	Not reached
Imatinib	Hindi et al. Eur J Cancer. 2015	retrospective	Advanced	Chordoma	46	9.9	30
Sorafenib	Bompas et al. Annals of oncology. 2015	Phase II	Advanced	Chordoma	27	Not reached (9 months PFS=73%)	Not reached (9 months OS=87%)
Dasatinib	Scheutze et al. Cancer. 2016	Phase II	Advanced	Ewing LMS Liposarcoma MPNST OSa RMS UPS	200	1.9	8.6
Pazopanib vs. Placebo	Kawai et al. Jpn J Clin Oncol. 2016	Phase III	Advanced	STS	47	1.6 vs. 5.7	15.4 vs. 14.9 (NS)
Pazopanib plus radiotherapy	Haas et al. Acta Oncol. 2015	Phase I	Preoperative for locally advanced	STS	10	NA	NA
Pazopanib	Ronellenfitsch et al. BMJ Open. 2016	Phase II	Preoperative for high risk early stage	STS	recruiting	TBD	TBD

Abbreviations: DFSP = dermatofibrosarcoma protuberans, LMS = leiomyosarcoma, MPNST = malignant peripheral nerve sheath tumor, OSa = osteosarcoma, RMS = rhabdomyosarcoma, UPS = undifferentiated pleomorphic sarcoma, STS = soft tissue sarcoma, NA = not available, TBD = to be determined, NS = not significant

### Imatinib

Imatinib is a tyrosine kinase inhibitor that inhibits several oncogenic pathways. Its impressive activity in sarcomas has been primarily shown in GIST, by interrupting the constitutive activation of KIT-mediated signal transduction characteristic of that sarcoma [79–81]. Imatinib has also demonstrated clinical benefit in the metastatic setting for dermatofibrosarcoma protuberans (DFSP) through inhibiting the activation of platelet derived growth factor receptor beta (PDGFR-β) [82,83]. DFSP tumors are characterized by PDGFB rearrangements (resulting in overexpression of PDGFβ), and 10-20% progress to a more aggressive, higher-grade subtype, designated as fibrosarcomatous transformation (FS-DFSP) [84–86]. Scarce data exists on the role of imatinib specifically in these metastatic FS-DFSP tumors. A recent retrospective study evaluating 10 metastatic FS-DFSP patients treated with imatinib showed that 90% of patients achieved clinical benefit (8 partial responses and 1 stable disease) with a median of 11 months PFS [87]. However, the duration of response observed in this study was lower than that reported ina previous series containing a mix of cases with both low-grade and FS-DFSP [88]. Moreover, this study showed that imatinib failed to eradicate the metastatic disease, as all five patients who had a complete resection of the residual tumor after imatinib still experienced a relapse. These findings suggest that despite a high expression of PDGFβ in DFSP, there might be other putative targets that contribute to disease progression and the development of imatinib resistance in metastatic patients. Interestingly, RNA transcriptional profiling of the study cohort revealed a simultaneous down-regulation of kinase signaling pathways and up-regulation of pathways involved in antigen processing and presentation [87]. These findings support the potential role for incorporating immune system enhancement strategies to achieve higher durable responses in the setting of imatinib-refractory FS-DFSP. Recently, CDK4/CDK6 inhibitors demonstrated pre-clinical activity against imatinib-resistant FS-DFSP [83].

The role of imatinib as a PDGFR-β inhibitor was also tested among metastatic chordoma patients, a disease displaying PDGFR-β protein expression but not amplification or other activating mutation. In a recent retrospective analysis that included 46 PDGFR-β-positive metastatic chordoma cases, median PFS was 9.9 months. Within a median follow up of 24.5 months, 34 of 46 of patients had stable disease by RECIST 1.0, with no partial or complete responses observed [89]. These results are consistent with previous findings reported in a phase II trial in 50 patients with advanced chordoma treated with imatinib (median PFS = 9 months and 70% of patients achieved stable disease) [90]. The very limited response rates observed in these trials, despite reportedly high expression of PDGFR-β, suggest a potential role for targeting related pathways other than PDGFR-β using other therapies. As such, the combination of imatinib and histone deacetylase inhibitor in recurrent chordoma patients is currently under evaluation (NCT01175109).

### Sorafenib

Sorafenib, a multi-kinase inhibitor, has been shown to act through different signal transduction pathways, including via inhibition of pro-angiogenic vascular endothelial growth factor receptors (VEGFR and PDGFR-β). In phase II clinical trials, sorafenib has been shown to have activity in metastatic soft tissue sarcoma [91], specifically in leiomyosarcoma [92]. Furthermore, a pre-planned exploratory analysis of a phase II clinical trial indicated that this agent has activity in angiosarcoma [93].

A recently published phase II trial evaluated the role of sorafenib as a PDGFR-β inhibitor in locally advanced and metastatic chordoma patients (n = 27) [94]. In this trial, the 9-month PFS was 73% and the 12-month OS was 86.5%. Compared to previous studies evaluating the role of imatinib in metastatic chordoma, 92% (12/13) of the assessable patients in this trial, who were not pre-selected based on PDGFR-β status, had a stable disease by RECIST criteria.

### Dasatinib

Dasatinib is a multi-kinase inhibitor that targets several oncogenes. Its main activity in sarcoma is thought to be through inhibiting the c-SRC kinase pathway. Gene expression profiling has reported the c-SRC pathway to be highly expressed in leiomyosarcoma [95] and chondrosarcoma [96]. Pre-clinical activity has been demonstrated in different cell lines, including rhabdomyosarcoma, osteosarcoma [97], Ewing sarcoma [97,98], and synovial sarcoma [99]. Despite this pre-clinical evidence, a large phase II clinical trial conducted by the SARC group, which included 200 patients from seven different cohorts of advanced sarcoma, showed low clinical benefit from dasatinib [100]. The study was terminated early for futility in five cohorts; only the cohorts of leiomyosarcoma and undifferentiated pleomorphic sarcoma fully accrued. However, dasatinib still did not show clinically-significant activity in these two cohorts, with only two objective responses observed in undifferentiated pleomorphic sarcoma patients [100]. Currently, the activity of dasatinib in combination with the CTLA4 inhibitor ipilimumab is being assessed in a phase I trial in unresectable or advanced soft tissue sarcoma (NCT01643278).

### Pazopanib

Pazopanib is a multi-kinase inhibitor that has been shown to have activity in metastatic soft tissue sarcoma, primarily through the phase III PALETTE trial published in 2012, which randomized metastatic soft tissue sarcoma patients to receive either pazopanib or placebo. This trial demonstrated a significantly prolonged PFS by 3 months in pazopanib arm, although no significant differences were observed in OS [101]. In a recent analysis on the Japanese subpopulation in PALETTE, pazopanib demonstrated results consistent with that observed in the PALETTE trial global population [102]. It should be noted that a pre-planned analysis on the original PALETTE trial did not reveal a superior benefit of pazopanib in specific sarcoma subtypes [101]. Moreover, a retrospective analysis limited to the uterine sarcoma cases from the PALETTE trial did not show significant activity of pazopanib against uterine sarcoma when compared to other subtypes [103].

Pazopanib has also been reported to be active against desmoid-type fibromatosis [104], which is being compared to chemotherapy in a phase II clinical trial (NCT01876082).

The role of pazopanib in the neoadjuvant setting was also investigated in several studies, based on recent evidence suggesting a synergistic effect when combining radiotherapy with angiogenesis-targeted therapies that act through inhibiting the supplying vasculature for sarcoma cells [105]. A recent phase I trial assessed the neoadjuvant combination of pazopanib and radiotherapy in locally advanced soft tissue sarcoma [106]. While none of the ten patients showed a volume reduction after radiotherapy, a high pathologic complete response rate (>95%) was observed in four patients. The overall findings of this trial showed that pazopanib and radiotherapy is tolerable in the neoadjuvant setting. Currently, a phase II/III trial (PAZNTIS) is assessing pre-operative chemoradiation or radiation with or without pazopanib for non-rhabdomyosarcoma soft tissue sarcomas (NCT02180867). Moreover, a study protocol recently published by the German Interdisciplinary Sarcoma Group described an ongoing phase II clinical trial (GISG-04/NOPASS) that is assessing the role of pazopanib in high-risk, resectable soft tissue sarcoma patients treated with radiotherapy [107]. Interestingly, in this trial, the primary endpoint is defined as metabolic response rate, measured as a reduction in the uptake value in post- versus pre-treatment using positron emission tomography (PET-CT) (NCT01543802).

### RANKL inhibitor (denosumab)

Denosumab is a monoclonal antibody that targets the receptor activator of nuclear factor-kappa b ligand (RANKL), which is normally expressed on osteoblasts and functions to activate osteoclasts to control bone regeneration and remodeling. This agent has exhibited a particularly significant clinical benefit in giant cell tumors, which highly express RANKL [108–111]. While the current treatment options available for this benign but locally aggressive bone tumor are mainly surgical, depending on site, this can be associated with severe morbidity [108,112]. However, a recent phase II trial conducted on 222 patients with technically-resectable giant cell tumors treated with neoadjuvant denosumab for a median duration of 15.3 months not only exhibited down-staging of the tumor, but also showed activity in restoring the bone with increased cortical thickness [113]. Moreover, 48% (106/222) of the patients in this trial either delayed their need for surgery or underwent less morbid interventions than had appeared necessary prior to denosumab treatment [113]. These promising results support the role of denosumab in achieving disease control and favorable clinical outcomes without exposing patients to potentially high-morbidity surgical interventions.

## EPIGENETIC THERAPY

### Epigenomics

Epigenetic and epigenomic modifications are emerging as key mechanisms in the pathogenesis of many sarcomas. Some subtypes could be described as exhibiting an “epigenomic mutator phenotype,” wherein a single missense mutation at an epigenetic modification target site, generates an “oncohistone,” resulting in aberrant repression and de-repression of genes at the global transcriptome level. An example of one such mutation, investigated by Lu et al [114], is seen in ~95% of chondroblastomas, in which a lysine-to-methionine mutation in histone 3.3 (H3.3K36M) has a dominant negative effect over the thirty other H3 histone alleles, inhibiting the normal methylation of wild-type H3K36. This results in aberrant expression of genes associated with mesenchymal differentiation, leading to the development not only of chondroblastoma, but also of some cases of undifferentiated sarcoma. These oncohistones not only reduced (activating) H3K36 methylation, but also increased (repressive) H3K27 methylation. Although the H3.3K36M mutation is specific to chondroblastomas [115], this study identified an H3.1K36M mutation in a pediatric undifferentiated soft tissue sarcoma, suggesting that K36M mutations in other H3 histones might play a role in poorly differentiated sarcomas. In follow-up papers investigating the mechanism by which K-to-M oncohistones inhibit global histone lysine methylation [116,117], it was found that the mutant histones occupy the active sites of histone methyltransferases, inhibiting their function and sequestering them from wild-type H3 histones.

In 2014, MPNST saw major breakthroughs in understanding the epigenetic mechanisms by which somatic mutations bring about malignant transformation, which have since led to practical diagnostic advances. Lee et al [118] identified loss-of-function mutations in EED and SUZ12, key components of the PRC2 polycomb repressive complex, in 12 of 15 (80%) cases of MPNST. Similarly, Zhang et al [119] conducted whole-genome sequencing on MPNSTs and found mutations critical to PRC2 functioning (SUZ12, EED, EZH2) in nearly half (24/50) of samples (alterations not seen in 11 sequenced neurofibroma samples). Given that the primary function of PRC2 is methylation of H3K27, these studies also identified complete loss of trimethylated H3K27 (H3K27me3) in samples with PRC2-related mutations. Lee et al [118] further showed that H3K27 trimethylation could be recovered in vitro by re-introducing the lost PRC2 component, which concomitantly decreased cell growth. This loss of H3K27 methylation was subsequently confirmed in three independent studies. Schaefer et al [120] evaluated immunohistochemistry for H3K27me3 in 100 MPNSTs and found that 51 (51%) were negative, correlating with higher grade. Among 200 MPNST mimics also evaluated, only 4 (2%) were negative for H3K27me3. Cleven et al [121] also performed H3K27me3 immunohistochemistry, and observed loss of H3K27me3 in 34% (55/162) of MPNSTs, while expression was retained in neurofibromas (n = 32). Within other tumors, H3K27me3 loss was seen in 24 of 341 (7%) cases, notably 9 of 15 (60%) synovial sarcomas and 3 of 8 (38%) fibrosarcomatous-DFSP. In this sample set, H3K27me3 loss correlated with inferior survival. Rorich et al [122] used DNA methylation arrays to characterize 171 peripheral nerve sheath tumors, and found that 21 of 41 (51%) MPNSTs had loss of H3K27 trimethylation. This study also confirmed PRC2 loss-of-function mutations among H3K27me3-deficient MPNSTs, identifying 15 (71%) and 4 (19%) cases with SUZ12 and EED mutations, respectively. Taken together, these studies show that loss of H3K27me3 is highly specific for MPNST and may be a useful diagnostic marker, particularly for its histologically-difficult distinction from neurofibroma, but not for distinction from malignant mimics synovial sarcoma and FS-DFSP. Further, loss of H3K27me3 was associated with higher grade and poorer survival in MPNST, so loss of PRC2 function may be contributing to increased tumor aggressiveness.

In rhabdomyosarcoma, the DNA methylome was characterized for 37 tumors and 10 cell lines [123], and PAX3-FOXO1 fusion-positive tumors showed distinctly lower global methylation than in fusion-negative tumors. Fusion-negative rhabdomyosarcoma bore more resemblance to normal skeletal muscle, suggesting that fusion-positive rhabdomyosarcoma has aberrant DNA methylation. Sites of abnormal methylation were associated with changes in mRNA expression patterns, and these sites included an augmented number of PAX3-FOXO1 target gene binding sites. This finding suggests that the rhabdomyosarcoma fusion protein modifies DNA methylation to regulate target gene expression.

### Translational science

#### Bromodomain

Bromodomain and extra terminal domain (BET) proteins are “readers” of histone acetylation marks, facilitating the transcription of genes in marked areas. In cancer, BET proteins are key translators of aberrant acetylomes, and as such, BET inhibitors are emerging as promising treatments for some cancers, including bone sarcomas.

In osteosarcoma, Lee et al [124] and Baker et al [125] investigated the use of BET protein BRD4 inhibitor JQ1. In vitro, JQ1 treatment inhibited cell line proliferation and survival; however, in vivo, JQ1 alone was ineffective against mouse cell line xenografts [124]. Combination with the mTOR inhibitor rapamycin was able to overcome resistance to JQ1 treatment in both models. Because BET inhibitors like JQ1 function by decreasing transcription of genes with aberrantly acetylated histones, the authors also explored changes in transcription induced by treatment. They identified RUNX2 [124] - an osteoblast differentiation transcription factor - and FOSL1 [125] - part of the AP-1 differentiation/proliferation/apoptosis transcription factor complex - as abnormally expressed genes that are directly modified by JQ1 treatment.

In Ewing sarcoma, BET inhibition was shown to directly block transcription of the fusion oncoprotein in two independent studies [126,127], which observed a strong down-regulation of EWS-FLI1 in cell lines following treatment with JQ1. Chromatin immunoprecipitation demonstrated that BRD4 becomes depleted in the fusion oncogene's promoter [127], and RNA microarray analysis revealed down-regulation of Ewing sarcoma-associated expression programs [126]. Cell line proliferation and migration were inhibited with JQ1 treatment, which induced both cell cycle arrest and apoptosis. In mouse xenografts, treatment suppressed tumor development. Taken together, these studies suggest that BET inhibition interferes with EWS-FLI1 activity and may prove a promising targeted strategy for treatment of Ewing sarcoma.

### EZH2

EZH2 is the catalytic subunit of the PRC2 transcriptionally repressive chromatin remodeling complex. Lv et al [128] found that EZH2 is overexpressed in osteosarcoma and correlates with poor prognosis. RNA silencing of EZH2 inhibited tumor growth in cell lines, induced apoptosis, and enhanced sensitivity to cisplatin. In vivo, EZH2 knockdown impaired xenograft growth and metastasis. These results suggest that EZH2 is important for tumor growth and metastasis in osteosarcoma, implying that EZH2 inhibition may prove a worthwhile treatment strategy as such drugs become available [129–133].

### Synovial sarcoma

Laporte et al [134] employed proximity ligation assay to validate the previously proposed [135] association of synovial sarcoma fusion oncoprotein SS18-SSX with TLE1 cofactor. TLE1 co-localized in the nucleus with SS18-SSX (but not with wild-type SS18) in cell lines and in human tumor tissue, and these interactions were disruptable by treatment with histone deacetlyase inhibitors. This technique could be useful for identifying agents that disrupt the oncoprotein complex in high-throughput drug screens for this disease. EZH2 inhibitors may also have activity in this disease [136,137].

### Clinical trials

#### HDAC Inhibitors

In pre-clinical studies, histone deacetylase (HDAC) inhibitors have been shown to be particularly active in translocation-associated sarcomas such as synovial sarcoma and Ewing sarcoma, through reversing aberrant transcriptional repression induced by the underlying fusion proteins in these sarcomas [135,138,139]. Clinical studies in other types of cancer have demonstrated an enhanced efficacy of HDAC inhibitors when combined with topoisomerase II inhibitors, such as anthracyclines, leading to more transcriptionally-active chromatin that is primarily observed with the administration of HDAC inhibitors prior to anthracyclines [140,141]. This observation was the basis for the design of a phase I trial assessing the HDAC inhibitor panobinostat followed by the topoisomerase II inhibitor of epirubicin in patients with advanced soft tissue sarcoma [142]. Among 20 patients included in this trial, 60% (n = 12) achieved a clinical benefit, suggesting that the combination of panobinostat and epirubicin might have value in overcoming anthracycline resistance. Currently, different epigenetic treatment strategies, including HDAC inhibitors, are being evaluated in a number of active sarcoma clinical trials (NCT01136499), (NCT01879085), (NCT00937495).

## IMMUNE THERAPY

### Immune microenvironment of sarcomas

The immune microenvironment of sarcomas is poorly characterized to date, leaving open the question of which sarcoma subtypes are immunogenic. D’Angelo et al [143] conducted an immunohistochemistry survey of 50 soft tissue sarcomas to evaluate the presence of tumor-infiltrating lymphocytes (TILs), tumor-associated macrophages, and immune checkpoint receptor and ligand, PD1 and PD-L1. Immunohistochemical staining examined CD3 (TILs), CD4 (helper T-cells), CD8 (cytotoxic T-cells), FOXP3 (regulatory T-cells), PD1, and PD-L1 expression, and multiplex IHC was performed for CD3/PD1, CD3/CD8, and CD3/CD4/FOXP3. The presence of macrophages was evaluated histologically. Lymphocyte and macrophage infiltration were observed in 98% and 90% of cases, respectively. Defining “low” or “high” density TILs as below or above 5%, they noted that 27 (54%) had low-density TILs, mainly leiomyosarcoma (3/4), synovial sarcoma (4/5), and chondrosarcoma (1/1), and 22 (44%) had high-density TILs, mainly GIST (9/14). Tumor, lymphocyte, and macrophage PD-L1 expression were 12%, 30%, and 58%, respectively, with the highest frequency of PD-L1 positivity seen in GIST (4/14). They observed no clear correlation between marker expression and clinical outcomes in this small study. Movva et al [18] also assessed PD-L1 expression by immunohistochemistry across 221 sarcomas, and found that 57% expressed PD-L1 and 54.8% had PD-1+ TILs. Significantly high expressors of PD-L1 included 19 of 60 (32%) leiomyosarcomas, 12 of 16 (75%) chondrosarcomas, 23 of 30 (77%) liposarcomas, and 7 of 10 (70%) undifferentiated pleomorphic sarcomas.

Smaller studies focusing on specific subtypes revealed broadly similar results. A study of 35 well- and de- differentiated liposarcomas [144] found TILs in all samples by flow cytometry, with a greater prevalence of CD4+ (80%) than CD8+ (20%) T-cells. Among CD8 T cells, 65% expressed PD-1. They also found mature dendritic cells in close proximity with CD4+ T-cells, suggesting intra-tumoral antigen presentation. Feng et al [145] examined 78 chordomas by immunohistochemistry and found that 75% have TILs present. While PD-L1 expression was seen in 95% of samples, 43% were classified as “PD-L1-high” due to moderate or strong staining intensities. While presence of these TILs correlated with PD-L1 expression, there was no clear correlation with survival. In osteosarcoma, Fritzsching et al [146] surveyed 135 samples for CD8+ and FOXP3+ T-cell presence by immunohistochemistry. 95% of cases had both CD8+ and FOXP3+ T-cells, and a high CD8:FOXP3 ratio correlated with improved survival. It is clear that the immune microenvironment of sarcomas is highly variable; however, given the strong immune presence in some subtypes, there is promise for immune therapy in many of these malignancies. Table 3 summarizes the clinical trials in immune therapy described below.

**Table 3:** A survey of recently published immune therapy studies in sarcoma

Immune Therapy	Study Analyzed	Design	Stage	Subtype	N	Median PFS (months)	Median OS (months)
Ipilimumab	Merchant et al. Clin Cancer Res. 2016	Phase I	Advanced	CCS OSa RMS SS	17	NA	NA
Pembrolizumab	Tawbi et al. ASCO Meeting Abstracts. 2016	Phase II	Advanced	CSa DDLPS Ewing LMS OSa SSUPS	40	Not reached	Not reached
Dendritic cell training, tumor lysate, reinfusion	Merchant et al. Clin Cancer Res. 2016	Phase II	Advanced	STS	29	24	42
Tumor cell transduction with GM-CSF, radiation, reinfusion	Goldberg et al. Clin Cancer Res. 2015	Phase I	Advanced	ASPS CCS	11	NA	NA
HER-2 expressing CAR-T-cells	Ahmed et al. J Clin Oncol. 2015	Phase I/II	Advanced	DSRCT Ewing OSa	16	1.5	10.3
MAGE-A1, MAGE-A3, and NY-ESO-1 dendritic cell vaccine (with decitabine)	Krishnadas et al. Cancer Immunol Immunother. 2015	Phase I	Advanced	Ewing RMS	2	0	NA

Abbreviations: PFS = progression-free survival, OS = overall survival, ASPS = alveolar soft part sarcoma, CCS = clear cell sarcoma, CSa = chondrosarcoma, DDLPS = dedifferentiated liposarcoma, DSRCT = desmoplastic small round cell tumor, LMS = leiomyosarcoma, OSa = osteosarcoma, RMS = rhabdomyosarcoma, SS = synovial sarcoma, STS = soft tissue sarcoma, UPS = undifferentiated pleomorphic sarcoma, NA = not available

### “Hot” tumors: checkpoint inhibitor strategies

Hot tumors are those that are immunogenic, associated with high numbers of TILs and tumor associated macrophages, but that are actively modulating the immune response to survive, for example by expressing immune checkpoint ligands that suppress anti-tumor immune responses. Hot tumors are the most likely to benefit from immunomodulatory therapies such as checkpoint inhibitors.

### Translational science

Lussier et al [147] showed that metastatic osteosarcomas (but not primary tumors) express PD-L1 and are infiltrated by PD1+ T-cells. Upon treatment with an anti-PD-L1 monoclonal antibody in a mouse cell-line xenograft model of metastatic osteosarcoma, they observed improved cytotoxic T-cell functioning, decreased tumor burden, and increased survival. However, in a follow-up study [148], they noted that xenografts quickly become resistant to PD-L1 blockade through the up-regulation of additional checkpoints, including CTLA-4. Treatment with a combination of anti-PD-L1 and anti-CTLA-4 monoclonal antibodies was able to control tumors completely and also conferred immunity to further tumor inoculation. Together, these pre-clinical results demonstrate that checkpoint blockade might be a worthwhile strategy for metastatic osteosarcoma patients.

### Clinical trials

Ipilimumab, an anti-CTLA-4 therapeutic monoclonal antibody, was tested in a phase I trial on 33 pediatric patients with refractory solid tumors, including 17 sarcomas (8 osteosarcomas, 2 rhabdomyosarcomas, 2 clear cell sarcomas, 2 synovial sarcomas) [149]. At 3 weeks, researchers observed increased numbers of circulating activated T-cells, predominately CD4+ helper T-cells. Toxicities were similar to those reported in adult patients. Although no objective tumor responses were observed, 6 subjects had stable disease at 6 weeks, including cases of osteosarcoma, clear cell sarcoma, and synovial sarcoma. Furthermore, subjects with immune-related toxicities exhibited improved overall survival over those who showed no evidence of immune stimulation.

In an abstract recently published for ASCO, Tawbi et al [150] presented the first results from SARC028, a much-anticipated phase II study of the PD1 inhibitor pembrolizumab in 40 soft tissue (leiomyosarcoma, dedifferentiated liposarcoma, undifferentiated pleomorphic sarcoma, synovial sarcoma) and 40 bone (osteosarcoma, Ewing sarcoma, chondrosarcoma) sarcomas. At 8 weeks, partial responses by RECIST 1.1 were observed for undifferentiated pleomorphic sarcoma (4/9), dedifferentiated liposarcoma (2/9), synovial sarcoma (1/9), chondrosarcoma (1/6), and osteosarcoma (1/19). Stable disease was observed in some cases for all subtypes enrolled. The PFS rate at 8 weeks was 50% in leiomyosarcoma, 63% in liposarcoma, 30% in synovial sarcoma, 67% in UPS, 24% in osteosarcoma, 9% in Ewing sarcoma, and 67% in chondrosarcoma. A new phase II study (NCT02500797) for ipilimumab (anti-CTLA-4) with or without nivolumab (anti-PD1) is currently accruing patients with unresectable or metastatic bone or soft tissue sarcoma. Together, these studies suggest select and limited use for checkpoint blockade in these diseases; however, because immune therapy trials are only just beginning for sarcomas, it is difficult to draw concrete conclusions from the evidence available.

### “Cold” tumors: immune augmentation strategies

Cold tumors are those that appear not to have been recognized by the immune system much - if at all - and theoretically would benefit best from stimulatory immune therapy such as cytokine treatment, immune cell engineering, and/or cancer vaccines.

### Translational science

Yang et al [151] investigated STAT3 inhibitors as an adjuvant to conventional chemotherapy. STAT3 is an oncogenic transcription factor that functions to enhance cell growth, inhibit apoptosis, and mediate immunosuppression. Knockout or pharmacologic inhibition of STAT3 in syngeneic fibrosarcoma tumors in mice enhanced growth inhibition by anthracycline-based chemotherapy. This response was only found in immunocompetent, not immunodeficient mice, likely due to immune activation from STAT3 inhibition. There was increased tumor infiltration by dendritic and cytotoxic T cells and an increase in the expression of interferon-responsive genes. Reintroduction of wild-type STAT3 inhibited this expression pattern and eliminated the improved response to chemotherapy. These pre-clinical results suggest that STAT3 inhibitors may improve the outcome of chemotherapy via activation of the immune system.

Dendritic cell strategies involve removal of a patient's own immune cells, dendritic cell training with or without tumor antigen exposure/engineering, and re-introduction of the trained dendritic cells. In osteosarcoma, dendritic cells from an osteosarcoma rat model were electrically fused with an osteosarcoma cell line to generate a fusion tumor vaccine [152]. This fusion vaccine was reintroduced, resulting in T lymphocyte proliferation. CD8+/CD4+ T-cell ratio increased, and CD4+ T-cell percentage dropped. Following vaccination, tumors shrunk or disappeared entirely, leading to improved survival of the rats. Also in osteosarcoma, Kawano et al [153] found that a combination of a dendritic cell strategy with an anti-GITR antibody inhibited tumor growth in a mouse xenograft model. Therapy constituted treatment with tumor lysate-pulsed dendritic cells, combined with an agonist for GITR, a co-stimulatory receptor for CD4/CD8 T-cell proliferation and effector functions. With combination treatment, they observed tumor growth inhibition and improved survival. Furthermore, this regimen increased the numbers of cytotoxic and reduced the numbers of regulatory T-cells. The same group found that combining doxorubicin with the same dendritic cell therapy in that murine osteosarcoma model induces immunogenic cell death and tumor inhibition [154]. This combinatorial approach is particularly appealing, as it suggests that dendritic cell therapy may serve to significantly enhance responses to conventional chemotherapy. Taken together, these studies suggest a promising future for dendritic cell therapies in osteosarcoma.

The innate immune system's natural killer (NK) cells are newer targets for immune therapy interventions. NKG2D is an activating immune receptor on NK and cytotoxic T cells whose ligands are frequently present on tumor cell surfaces but rarely detectable on normal cells. Fernández et al [155] found moderate to high levels of NKG2D ligand expression by flow cytometry on all of 22 human osteosarcoma cell lines. Using a mouse osteosarcoma xenograft model injected with human natural killer cells, they showed that treatment of mouse osteosarcoma xenografts with IL-2 and the diuretic spironolactone resulted in enhanced receptor-ligand interactions of NKG2D, leading to NK cell activation, expansion, and targeting of osteosarcoma tumor-initiating cells. Zhu et al [156] found that osteosarcoma expression of NKG2D receptor and ligands were enhanced by treatment with the HDAC inhibitor entinostat, an effect not seen in normal human fibroblasts. In a mouse cell line xenograft model, mice treated with both entinostat and NK cells had a significantly reduced tumor burden, supporting the hypothesis that HDAC treatment sensitizes osteosarcoma cells to NK-mediated cell death. Finally, Jemitzky et al [157] show that anti-IGF1 receptor therapeutic antibodies not only diminish Ewing cell line viability, but also promote in vitro expansion of human NK cells. When co-incubated with Ewing sarcoma cell lines, NK cells exhibit potent degranulation responses. These studies together suggest that combining adoptive transfer of activated NK cells with IL-2, HDAC, anti-IGF1R, or other agents may have therapeutic benefit in sarcomas.

### Clinical trials

A phase II cancer vaccine trial for 29 sarcomas by Merchant et al [158] employed a particularly complex protocol involving autologous monocyte extraction, dendritic cell training, tumor lysate-pulsing, and reinfusion. They observed a 62% 5-year overall survival, which is a significant improvement over the subjects’ expected 25% survival (based on previous studies, not a randomized control group). Benefits were seen chiefly in Ewing sarcoma and rhabdomyosarcoma (63%), whereas no benefit was seen in synovial sarcoma or desmoplastic small round cell tumor (0%). T-cell responses were identified in 62% subjects, and these responses were associated with improved survival.

In a phase I trial for alveolar soft part sarcoma and clear cell sarcoma by Goldberg et al [159], tumor cells from subject metastases (n = 11) were transduced with GM-CSF, irradiated, and re-infused. Vaccination enhanced infiltration of local dendritic cells and stimulated T cell reactions to tumor cells. Tumor biopsies showed PD1-positive T cells and PD-L1-expressing sarcoma cells, in correlation with each other. No tumor regressions were observed, but authors suggest concomitant treatment with checkpoint blockade to improve antitumor immunity.

Immune therapy against specific tumor antigens may prove a particularly useful strategy in sarcomas, due to their comparatively low genomic complexity (and presumed low burden of tumor neoantigens). Chimeric antigen receptor T-cells (CAR-T-cells) are T-cells, usually autologous cells extracted from the patient receiving therapy, that have been engineered to target a specific tumor antigen. Ahmed et al [160] conducted a phase I/II trial using CAR-T-cells targeting HER-2-expressing sarcomas. At 6 weeks, stable disease was attained in 3 of 14 osteosarcomas, 1 of 1 desmoplastic small round cell tumor, and 0 of 1 Ewing sarcoma. No partial or complete responses were observed. They analysed peripheral-blood mononuclear cells to confirm presence of HER2-CAR-T-cells, which were detected in 14 of 16 treated patients. In a related phase I veterinary trial, 18 dogs presenting with osteosarcoma were treated with HER2 Listeria vaccine [161]. Treatment induced a HER2-specific interferon-γ response 15 of 18 dogs within 6 months. Additionally, they saw reduced rates of metastasis and improved 1-, 2- and 3- year survival rates, compared to a historical control group treated with amputation and chemotherapy alone.

Dendritic cell vaccines can also serve to target specific tumor antigens, as seen in a phase I trial by Krishnadas et al [162] in children with relapsed or refractory solid tumors, including Ewing sarcoma (n = 2), osteosarcoma (n = 2), and rhabdomyosarcoma (n = 1). The protocol involved treatment with decitabine (a cytotoxic therapy), followed by a vaccine of autologous dendritic cells pulsed with peptides derived from tumor antigens MAGE-A1, MAGE-A3, and NY-ESO-1. Only one Ewing sarcoma patient and one rhabdomyosarcoma patient received the dendritic cell vaccine, and while neither had any objective response to therapy, both developed an antigen-specific response in their CD4+ T-cells. Given the limited number of sarcoma subjects in this study and the presence of a tumor-specific immune response, this strategy warrants further exploration for these and other sarcoma subtypes, such as myxoid liposarcoma, in which a strong correlation between poor prognosis and high expression (protein and mRNA) of PRAME and/or NY-ESO-1 has been reported [163].

## SUMMARY

Recent publications have shown that significant, albeit incremental progress can still be made using cytotoxic chemotherapy agents, including eribulin, trabectedin, and aldoxorubicin. Efforts to conduct larger international studies have been somewhat successful, and there has been a partial move toward more histology-specific studies rather than the lumping together of completely disparate entities. Targeted therapies have shown fewer advances at the level of clinical practice, despite numerous trials involving various receptor tyrosine kinase inhibitors; however, advances in basic science, driven by spectacular technological advances in genomics, highlight targetable pathways, including mTOR, Notch, Wnt, Hedgehog, and MDM2, and other targetable proteins, such as Hsp90, RNA helicase, and FOXM1. Epigenetic approaches have had some preclinical success with BET and EZH2 inhibitors, and immuno-oncology approaches are advancing from pre-clinical to phase I- and II- level studies in both immunostimulatory and immune checkpoint therapies.
